# Emerging Lab-on-a-Chip Approaches for Liquid Biopsy in Lung Cancer: Status in CTCs and ctDNA Research and Clinical Validation

**DOI:** 10.3390/cancers13092101

**Published:** 2021-04-27

**Authors:** Ângela Carvalho, Gabriela Ferreira, Duarte Seixas, Catarina Guimarães-Teixeira, Rui Henrique, Fernando J. Monteiro, Carmen Jerónimo

**Affiliations:** 1 i3S-Instituto de Investigação e Inovação em Saúde, Universidade do Porto, Rua Alfredo Allen 208, 4200-135 Porto, Portugal; up201505325@fe.up.pt (G.F.); up201406215@fe.up.pt (D.S.); fjmont@ineb.up.pt (F.J.M.); 2 INEB-Instituto de Engenharia Biomédica, Universidade do Porto, Rua Alfredo Allen 208, 4200-135 Porto, Portugal; 3 Porto Comprehensive Cancer Center (P.CCC), R. Dr. António Bernardino de Almeida, 4200-072 Porto, Portugal; catarina.guimaraes.teixeira@ipoporto.min-saude.pt (C.G.-T.); rmhenrique@icbas.up.pt (R.H.); carmenjeronimo@ipoporto.min-saude.pt (C.J.); 4 Cancer Biology and Epigenetics Group, IPO Porto Research Center (GEBC CI-IPOP), Portuguese Oncology Institute of Porto (IPO Porto), R. Dr. António Bernardino de Almeida, 4200-072 Porto, Portugal; 5 Department of Pathology, Portuguese Oncology Institute of Porto (IPO Porto), R. Dr. António Bernardino de Almeida, 4200-072 Porto, Portugal; 6 Department of Pathology and Molecular Immunology, Institute of Biomedical Sciences Abel Salazar, University of Porto (ICBAS-UP), Rua Jorge Viterbo Ferreira 228, 4050-513 Porto, Portugal; 7 Faculdade de Engenharia, Departamento de Engenharia Metalúrgica e Materiais, Universidade do Porto, Rua Dr Roberto Frias, s/n, 4200-465 Porto, Portugal

**Keywords:** lung cancer, circulating biomarkers, liquid biopsies, microfluidic devices, CTCs, ctDNA

## Abstract

**Simple Summary:**

Lung cancer (LCa) remains the leading cause of cancer-related mortality worldwide, with late diagnosis and limited therapeutic approaches still constraining patient’s outcome. In recent years, liquid biopsies have significantly improved the disease characterization and brought new insights into LCa diagnosis and management. The integration of microfluidic devices in liquid biopsies have shown promising results regarding circulating biomarkers isolation and analysis and these tools are expected to establish automatized and standardized results for liquid biopsies in the near future. Herein, we review the status of lab-on-a-chip approaches for liquid biopsies in LCa and highlight their current applications for circulating tumor cells (CTCs) and circulating tumor DNA (ctDNA) research and clinical validation studies.

**Abstract:**

Despite the intensive efforts dedicated to cancer diagnosis and treatment, lung cancer (LCa) remains the leading cause of cancer-related mortality, worldwide. The poor survival rate among lung cancer patients commonly results from diagnosis at late-stage, limitations in characterizing tumor heterogeneity and the lack of non-invasive tools for detection of residual disease and early recurrence. Henceforth, research on liquid biopsies has been increasingly devoted to overcoming these major limitations and improving management of LCa patients. Liquid biopsy is an emerging field that has evolved significantly in recent years due its minimally invasive nature and potential to assess various disease biomarkers. Several strategies for characterization of circulating tumor cells (CTCs) and circulating tumor DNA (ctDNA) have been developed. With the aim of standardizing diagnostic and follow-up practices, microfluidic devices have been introduced to improve biomarkers isolation efficiency and specificity. Nonetheless, implementation of lab-on-a-chip platforms in clinical practice may face some challenges, considering its recent application to liquid biopsies. In this review, recent advances and strategies for the use of liquid biopsies in LCa management are discussed, focusing on high-throughput microfluidic devices applied for CTCs and ctDNA isolation and detection, current clinical validation studies and potential clinical utility.

## 1. Introduction

Lung cancer (LCa) is the leading cause of cancer-related death in both genders, worldwide. In 2020, LCa accounted for more than 2.2 million new cases, with the highest mortality rate, estimated to 1.8 million deaths [1].

Most cases correspond to non-small-cell lung cancer (NSCLC), with about 15% representing small-cell lung cancer (SCLC). Prognosis is unfavorable for both, with five-year survival rate under 20% and 5% for NSCLC and SCLC, respectively. Absence of specific symptoms at early stages of the disease contribute to the poor outcome. Indeed, most LCa are diagnosed at advanced stages, in which therapeutic strategies are less effective [2,3].

Tentative LCa screening may be performed using low-dose computational tomography scan (LDCT), followed by tissue biopsy to assess suspicious nodules, if present. Tissue biopsy remains the gold-standard for histopathological diagnosis and molecular characterization [4,5]. However, tissue biopsy might be challenging due to tumor inaccessibility or insufficient collection of tissue for diagnosis and molecular testing, not allowing for assessment of tumor heterogeneity. Since it is an invasive procedure, follow-up can be limited and poorly suited for patients with metastatic disease. Complications are frequently reported, with the most common being pulmonary hemorrhage and pneumothorax [6,7].

Minimally invasive liquid biopsies may easily overcome these limitations and complement tissue biopsies, providing real-time patient monitoring by following disease evolution and response to treatment. The principle underlying liquid biopsies is that biomarkers shed from the tumor and metastasis, can be retrieved from peripheral fluids such as blood. Circulating tumor cells (CTCs), cell free DNA containing circulating tumor DNA (cfDNA, ctDNA), extracellular vesicles (EVs), cell free microRNAs, and tumor educated platelets (TEP) are among the circulating biomarkers considered to contain tumor information [8,9,10]. In the past decade, research on liquid biopsies for LCa has significantly increased, becoming a non-invasive complement to the traditional tissue biopsies, and leading to several clinical validation studies, mostly focused on CTCs and ctDNA [11,12,13].

**Figure 1:**
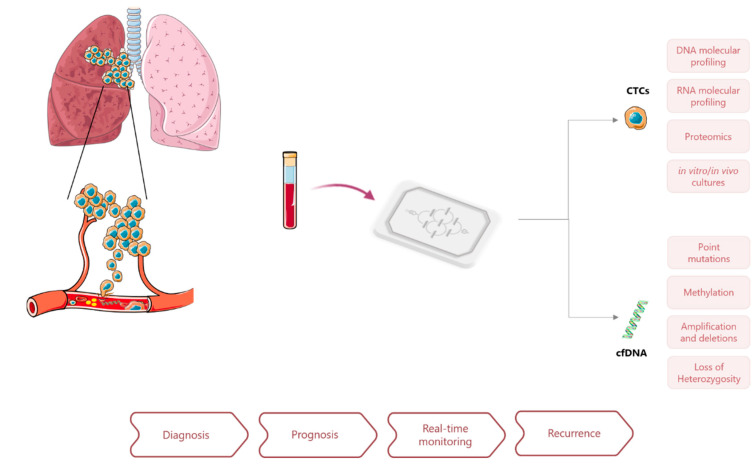
Application of microfluidics approaches for liquid biopsy in lung cancer and potential clinical applications of CTCs and ctDNA analysis for cancer diagnosis and management.

Microfluidic devices have been introduced in the field of liquid biopsies as a strategy to improve biomarkers separation, capture, and characterization (Figure 1). Advances in integrated microfluidics platforms have made possible the miniaturization of analytical techniques on-chip to enable accurate and high-throughput assays in a more automated manner. Other advantages relate to the low-cost of their production, the precise control of working parameters, closed architectures that prevent sample loss and operation with low volumes, and reduced use of reagents and waste production. Lab-on-a-chip approaches to liquid biopsy have shown promising applications for disease monitoring and genetic characterization of LCa in a minimally invasive way and with minute sample requirements [13,14,15,16].

Herein, we review the current microfluidics-based strategies applied in CTCs and ctDNA isolation and detection, with a special focus in research in LCa. Moreover, current clinical validation studies and trials, addressing the clinical utility that lab-on-a-chip approaches may provide to improve the diagnosis, prognosis, and monitoring of lung cancer evolution, response to treatment, and recurrence are critically reviewed.

## 2. Non-Small Cell (NSCLC) and Small Cell (SCLC) Lung Cancer

Based on the cell origin, lung cancer can be classified into two major subtypes: SCLC, which accounts for about 15% of all cases and NSCLC which represents 85% of total cases. NSCLC is further classified into three main different histological types—adenocarcinoma, squamous cell carcinoma, and large cell carcinoma—although many other rare entities exist [17]. In NSCLC, it is estimated that activating mutations of epidermal growth factor receptor (EGFR) occur in 10% to 20% of Caucasian and at least 50% of Asian cases, with the most common EGFR mutations including deletion in exon 19 (19 Del) or point mutation in exon 21 (L858R). Targeted therapies have been developed using tyrosine kinase inhibitors (TKIs) for treatment of patients harboring EGFR mutations. After the first-line treatment, a secondary resistant mutation (T90M) often occurs, estimated to affect 48–62% of EGFR TKI-resistant patients, to whom third-generation TKI may be administered [18,19]. Thus, early detection of activating and resistant EGFR mutations may allow for personalized treatment of NSCLC patients and improve clinical outcome [19].

Other identified mutations such as anaplastic lymphoma kinase (ALK) or ROS proto-oncogene 1 (ROS1) rearrangements also constitute therapeutic targets in NSCLC patients. Like EGFR TKIs, anti-ALK TKIs have also been developed, with multiple generations which tackle the acquisition of resistance mutations by cancer cells [20]. Mutations in other genes such as KRAS proto-oncogene GTPase (KRAS), tumor protein p53 (TP53), and phosphatidylinositol-4,5-bisphosphate 3-kinase catalytic subunit α (PIK3CA) are also reported in NSCLC, with co-existing mutations implicating lower progression free survival (PFS) [21]. The development of therapy resistance is associated with tumor heterogeneity and decisively contributes to poor overall survival (OS) in NSCLC.

A recent progress in the treatment of NSCLC has been immunotherapy. Programmed death 1 (PD-1) receptor and its ligand (PD-L1) have been shown to regulate antitumor responses. PD-L1 binding to PD-1 inhibits T-cell function and proliferation, resulting in ineffective immune response. Immunotherapy uses monoclonal antibodies that block PD-1/PD-L1 binding, thus favoring an active immune response. The use of PD-1/PD-L1 inhibitors has been shown to improve OS of NSCLC patients in several clinical trials and is part of current clinical practice [22,23].

Small-cell lung cancer (SCLC) is an aggressive tumor of neuroendocrine origin that metastasizes rapidly and is associated with poor outcome. Its staging comprises limited (LS) and extensive (ES) stage disease with the later accounting for nearly 65% of diagnosed cases. In recent years, the median OS has been estimated to be 8–12 months for patients with ES and 12–20 months for patients with LS [2,24]. Chemotherapy remains standard treatment in SCLC and has remained mostly unchanged for more than 30 years. Although SCLC is initially sensitive to treatment, relapse follows with acquired chemoresistance and yields poor responses of short duration [25,26]. Due to its close association with tobacco-derived carcinogens, SCLC discloses a highly heterogeneous genomic landscape with high somatic mutation burden. SCLC is also characterized by high incidence of inactivating mutations of TP53 and retinoblastoma 1 gene (RB1). Additional mutations have been detected at lower frequencies and in smaller populations of SCLC patients, such as amplification of MYC, MYCN, MYCL1, and FGR1 or inactivation of PTEN [25,27,28].

Unlike NSCLC, for which recent advances have led to the development of targeted therapies, SCLC has limited treatment options and effective druggable targets have yet to be identified. Most recently, immunotherapy targeting the programmed death ligand 1 (PD-L1) and CTLA4 in T cells has been investigated in the context of SCLC, with a few ongoing clinical trials. Two recent phase III trials which combined a PD-L1 inhibitor with standard chemotherapy improved the OS in patients with extensive disease. Thus, identification of biomarkers to select patient subgroups that may benefit from this combined strategy are urgently needed [29,30].

A substantial limitation in SCLC research and treatment has been the difficulty in recovering tumor tissue for analysis, which often consist of small samples, with inadequate quality, and re-biopsies are rarely performed in recurrent disease [28,31]. Tissue biopsy limitations also remains an obstacle for NSCLC treatment. When patients cannot undergo several biopsies, assessing tumor evolution according to treatment is impaired, limiting the follow-up of TKI-based treatments and acquired resistance mechanisms.

Liquid biopsies have already proved to be efficient for assessing tumor mutations in SCLC and NSCLC as acquired resistance mechanisms [28,32,33]. Liquid biopsies have the potential to reveal spatial and temporal tumor heterogeneity. Besides assisting in diagnosis and prognosis of LCa, real time-monitoring of therapies by following the mutational burden and detect minimal residual disease (MRD), a key advantage of liquid biopsies, may enable early detection of cancer in high-risk patients, for which existing screening methods are limited. Hence, highly sensitive and specific standardized techniques are needed. In this context, microfluidic-based strategies for liquid biopsy might be of significant clinical value [34,35].

## 3. Circulating Cancer Biomarkers

### 3.1. Circulating Tumor Cells (CTCs)

CTCs play a vital role in cancer progression, having a significant participation in the complex cancer metastatic process. When detached from the primary tumor tissue, these tumor-derived cells enter the bloodstream until reaching a potential site to migrate, where they extravasate from blood vessels and, if meeting an adequate microenvironment, can form metastasis [36]. As metastasis represent the main cause of death in cancer patients, the study of CTCs presence in blood and their properties is essential. Information gathered through detection and readout of these cells can serve as a tool for cancer screening, tumor diagnosis, development, and monitoring of personalized anti-cancer therapy efficacy [37,38,39].

CTCs face some challenges to colonize distant sites. Once in the bloodstream, they are attacked by immune cells and need to trespass vessels walls and survive as tumor-initiating seeds in host tissues. Therefore, only an extremely low portion of them survive and become available in peripheral blood. It has been estimated that, on average, there are about 1 to 10 cells per mL of blood, numbers that vary according to the type of cancer, disease stage, or ongoing treatment [5,6,40,41].

This rare event in blood, associated with cells’ heterogeneity, are the main limitations for approaches devised to capture and isolate tumor cells [42,43]. CTCs can travel throughout the blood individually or as clusters, which are considered to have more metastatic power (100-fold) than single CTCs [44]. Although in-depth knowledge about these clusters is still lacking, these aggregates seem to exhibit additional survival advantage and proliferation capabilities, being strongly associated with worse clinical outcome. Considered tumor microemboli, compared to single CTCs, clusters also seem to be less sensitive to chemotherapy [45,46,47].

Regarding LCa, CTCs have specific availability in blood depending on the cancer type, NSCLC or SCLC. In NSCLC, the concentration of CTCs can be low and difficult to assess at early stages, and due to the high percentage of LCa patients harboring NSCLC, it currently draws more attention. Several studies have investigated the prognostic value of CTCs in NSCLC. Lindsay et al. observed that, among 125 stage IIIB-IV NSCLC patients, more than 5 total CTCs per 7.5 mL of peripheral blood was associated with significantly reduced OS but not progression-free survival (PFS) [48]. Similarly, Qi and Wang also defined 5 CTCs per 7.5 mL of blood as cut-off when assessing 100 stage III lung squamous cell carcinoma patients. In their study, increased CTC count was an independent predictor for both OS and PFS [49].

On the other hand, SCLC patients can have ten times more CTCs in their bloodstream than patients with any other type of cancer and between 70% and 95% of patients have detectable CTCs, underlining SCLC aggressiveness, as the number of CTCs can be directly correlated with the disease stage and its clinical impact. A correlation between the presence of CTCs, PFS, and OS has already been reported [50,51,52]. Huang et al. reported on the utility of CTCs for the management of extensive stage SCLC. The study enrolled 26 chemo naïve SCLC patients that were analyzed prior to and after the therapy, at 6- and 8-week follow-up, as well as at relapse. Overall CTC detection in 24 patients was 75 (0–3430 range) at baseline and 2 (0–526 range) post-treatment. Additionally, higher CTCs baseline and percentage change after treatment were associated with decreased OS [51].

Recently, Syrigos et al. reviewed the existing studies on the prognostic role of peripheral CTCs in lung cancer (both NSCLC and SCLC). Despite variability among different investigations, such as the method used for cell isolation, disease subtype, patient stage, and CTCs cut-off value, most studies demonstrated that CTC quantification is strongly associated with survival, emphasizing its value for LCa prognostication. Furthermore, the need of standardizing assays to ensure consistent and reproducible results as well as to assess the diagnostic accuracy and cost-effectiveness of the techniques in prospective studies was highlighted [53].

CTCs can differ in size, number and protein expression, adding another layer of tumor heterogeneity. LCa-derived CTCs are highly heterogeneous, which strongly contributes to evasion from certain detection technologies. Heterogeneity arises from different parameters: epithelial-mesenchymal transition (EMT) process, cellular size, intact or apoptotic state, and the presence of somatic oncogenic mutations, commonly affecting EGFR and TP53, and ALK rearrangements [54]. The lack of tools to distinguish apoptotic cells from unnucleated or necrotic cells or circulating cell fragments also impair their isolation. Therefore, highly sensitive approaches that can standardize isolation and enumeration of CTCs may greatly improve the outcome of CTCs research in LCa.

### 3.2. Circulating Tumor DNA (ctDNA)

Circulating cell-free DNA (cfDNA) available in the bloodstream derives from passive and active release by cells. In cancer patients, circulating tumor DNA (ctDNA), represents a fraction of total cfDNA and has been explored for cancer detection and as a prognostic and predictive marker. This circulating nucleic acid is believed to be shed from apoptosis, necrosis, or by active secretion of tumor cells from the primary tumor, metastatic lesions, or CTCs. ctDNA is highly fragmented and its short length (<200 bp) suggests an association with nucleosomes and release into circulation mainly through apoptosis [8,10,55,56]. Cancer patients disclose higher and more variable concentration of both ctDNA and non-tumor cfDNA, compared to healthy donors. Cancer subtypes and stage are two prominent variables of ctDNA levels. Other factors relate to tumor size, metabolism, and proliferation rate, which have been shown to correlate with the amount of DNA in plasma [57,58]. Interestingly, Bettegowda et al. showed, in several cancers, that stage IV ctDNA plasma concentration levels were 100-fold higher compared to stage I tumors and that detection levels also increased, with stage IV ctDNA detection levels at 82%, and stage I at 47% [59]. Tumor burden, accessibility to circulation due to tumor’s vascularization, and cell turnover also impact on ctDNA levels in circulation.

The analysis of ctDNA in clinical settings still faces some challenges such as low concentration and the distinction from cfDNA to accurately detect rare mutations. In fact, considering this limitation, many studies commonly report on cfDNA analysis, rather than ctDNA. Additionally, the variability of circulating nucleic acids concentration hinder the definition of a range value for ctDNA detection [55,59,60,61]. Nonetheless, circulating DNA proves to be crucial in producing an invaluable diagnosis and prognosis analysis through detection of relevant genetic alterations in real-time, such as point mutations and gene amplification, deletion, insertion, fusion, as well as epigenetic alterations (e.g., DNA methylation). Other enthralling aspects around this non-invasive biomarker involve its potential in monitoring therapy effectiveness by its quantitative and qualitive changes. ctDNA levels evaluation, as well as tracking of therapy efficacy by detecting acquired resistance mutations may allow for the assessment of disease progression, MRD, and predict relapse early on [35,62,63].

Oxnard et al. demonstrated the ability of liquid biopsies to track resistance mechanisms in NSCLC. In their study, 31% of patients with T790M-negative tissue samples, tested positive for T790M mutation in plasma. Objective response rate (ORR) and PFS were similar for patients with T90M detected in tumor or plasma, when treated with the third-generation EGFR-TKI Osimertinib. Plasma cfDNA analysis of T790M relative allele frequency could be critical as it informs on whether T790M is a dominant mechanism of resistance or a subclonal phenomenon with a heterogeneous biology [64]. In SCLC, Almodovar et al. first reported on a liquid biopsy assay to quantify somatic variants in cfDNA with a custom SCLC-specific gene panel. Disease-associated mutations were detected in 85% of patient samples with mutant allele frequencies ranging from 0.1% to 87%. In 59% of patients with ES SCLC, the most common mutations were detected in TP53 (70%) and RB1 (52%). Alterations in other ten genes (PTEN, NOTCH1, NOTCH2, NOTCH3, NOTCH4, MYC, MYCL1, PIK3CA, KIT, and BRAF) were also observed. Results showed that analysis of cfDNA provided evidence of disease relapse before conventional imaging in several cases [28]. Despite these compelling results, research on SCLC cfDNA analysis is still limited and more prospective studies are necessary.

Aberrant DNA methylation, commonly found in cancer and leading to dysregulated gene expression, has shown as a promising biomarker assessed in ctDNA. DNA methylation is essential for many biologic processes and, in cancer, altered methylation (hyper/hypomethylation) of CpG dinucleotides located at promoter frequently occurs. Methylated genes found in cfDNA have been used to distinguish not only LCa from benign conditions, but also to discriminate among LCa subtypes [65]. Blood-based liquid biopsy analysis disclosed that APCme, RARβ2me, RASSF1Ame, SEPT9me, and SOX17me could be used to detect LCa, with high specificity, and discriminate from benign lesions [66]. HOXA9 and RASSF1A genes were reported to be hypermethylated in SCLC, disclosing 64% and 52% sensitivity and 84 and 96% specificity, respectively, for cancer detection. Moreover, DCLK1me was more frequent in SCLC, and SHOX2me identified SCLC with 80% sensitivity, performing better than for lung squamous cell carcinoma or adenocarcinoma (63 and 39%). On the other hand, SEPT9me was more frequent (53%) in NSCLC as opposed to SCLC (26%) [65,67]. Although demonstration of the value of DNA methylation biomarkers still requires large-scale validation, it may constitute a major early detection marker for SCLC and NSCLC alike.

## 4. Lab-on-a-Chip Approaches for Liquid Biopsies

### 4.1. Microfluidic-Assisted Strategies in CTCs Research

**Figure 2:**
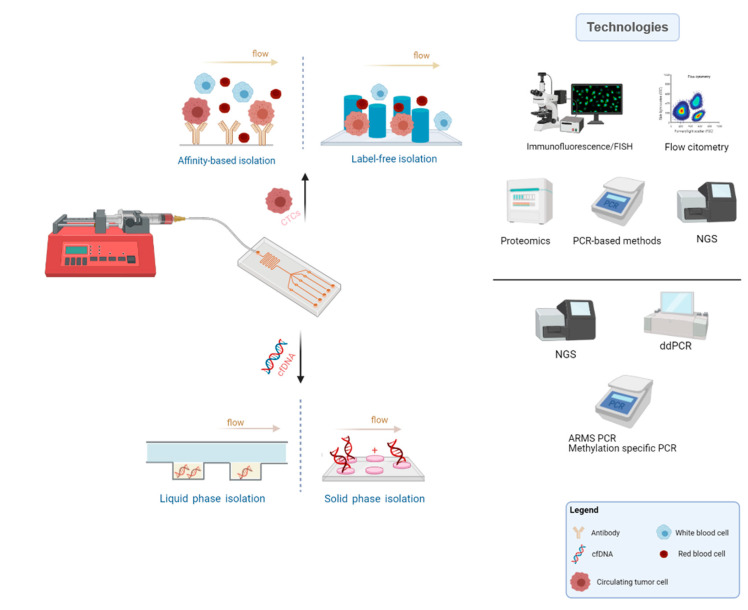
Common microfluidics methodology, main principles applied for CTCs and cfDNA isolation and typical technologies used for the biomarkers characterization (Created with BioRender.com).

There are numerous approaches for CTC enrichment and isolation relying on diverse principles such as dielectrophoresis, biofunctionalized magnetic beads, sized-dependent filters, and density gradient centrifugation. Nonetheless, among these, microfluidic chips stand out by their unique properties, easy manipulation, and suitable cell separation [68]. Many research groups have been developing microfluidic platforms for CTC enrichment, relying on the unique features of these tumor-derived cells and background blood cells. While some depend on biochemical properties and biological signature of CTCs (affinity-based strategies), others are constructed based on their biophysical properties (label-free strategies), compared to blood cells (Figure 2) [68,69,70,71]. A thorough review regarding technologies for CTCs enrichment has recently been published by Rushton et al. [72].

Separation based on biochemical properties of CTCs depends on specific bonding between tumor-specific antigens on the surface of cells and complementary ligands added and fixed along the microfluidic platform. Most of the available immune-based microfluidic systems utilize positive selection of CTCs, and rely on epithelial cell adhesion molecule, EpCAM, a transmembrane glycoprotein, expression. EpCAM is a surface marker commonly used to detect CTCs, since it is present in tumor cells derived from human epithelial tumors, but absent in blood cells [70,73]. CTC-chip, a microfluidic device for CTCs detection containing microposts coated with antibodies against EpCAM molecule was first introduced by Nagrath et al. The chip detected CTCs in 115 of 116 (99%) samples from patients with metastatic breast, colon, lung, prostate, and pancreatic cancer with 50% purity and a range of 5–1281 CTCs per sample ml of blood [74].

Maheswaran et al. reported on its application for the detection of the EGFR mutation in NSCLC patients by combining with the allele-specific Scorpion Amplification Refractory Mutation System (SARMS) assay. CTCs were detected in 27 patients with metastatic NSCLC using the CTC-chip. The activating mutation was found in 11 out of 12 patients with corresponding tissue analysis, and the T90M mutation was detected in 2 of 6 patients which showed response to TKIs and 9 of 14 who presented disease progression. Continuous analysis demonstrated a correlation between increasing number of CTCs with tumor progression and reduction with imagiological response [75].

NanoVelcro is another microfluidic strategy based on affinity-dependent isolation of CTCs, currently in its 4th generation. The system was originally composed by a silicon nanowire substrate (SiNS) combined with anti-EPCAM for CTCs capture and enumeration. Since then, it has evolved to strategies with improved CTCs purification and recovery by employing thermo-responsive substrates (3rd generation) and via surface chemistry with competitive binding (4th generation) allowing for capture and release of CTCs. The 4th generation NanoVelcro chip has shown CTC capture with well-preserved RNA transcripts in patients with prostate cancer [76,77].

Binding between anti-EpCAM antibodies coated throughout the microfluidic channels and EpCAM molecules present on cell’s surface, while improving isolation purity, only occurs in part of the available CTCs, i.e., EpCAM-positive tumor cells. Whereas most LCa subtypes originate from epithelial cells, cancer tumor cells are known to undergo EMT, thus possibly having downregulated EpCAM expression. Hence, EpCAM-based approaches may fail to detect a proportion of CTCs having little or no expression of this molecule, achieving false-negatives. Typically, cell surface markers associated with the epithelial phenotype—EpCAM and cytokeratin (CK)—and mesenchymal surface markers such as vimentin (VIM, a marker of EMT) and CD133, can be employed for LCa CTCs enrichment [54,78,79].

Antibodies for tumor-specific membrane antigens typically found in specific cancer types are needed to detect or to aid in CTCs quantification. For example, antibodies against tyrosine-protein kinase receptor (HER2), a common antigen in breast cancer cells, are used for CTC enrichment and isolation of CTCs from breast cancer patients [80]. Hyaluronic acid (HA) receptor CD44, has been proposed as LCa biomarker for capturing CTCs. This receptor is expressed in LCa cells, whereas background blood cells have reduced or no expression and poor adhesion to HA [81].

Alternatively, as tumor cells exhibit inter- and intratumor heterogeneity, some strategies perform CTC enrichment by depletion of leukocytes, typically using anti-CD45 antibodies. As they only interact with non-cancer cells, these approaches offer an opportunity to isolate CTCs regardless of their phenotype and ensure that CTC’s viability is maintained. Bu et al. reported an anti-CD45 immunoaffinity-based dual patterned immunofiltration (DIF) device for negative enrichment of tumor-cells. The dual pattern layer of the device improves the changes of contact between CD45 antibody and CTCs, enhancing results. A NSCLC cell line was used in erythrocyte-removed blood samples to test the performance of DIF system, with a 97% depletion of leucocytes and less than 10% of tumor-cells adhered to device´s surface through non-specific binding [82].

Contrarily to the aforementioned techniques, label-independent strategies for CTC separation do not rely on surface markers expression, which can be changeable, but on inherent biophysical properties within cancer cells and remaining blood cells, mainly size, compressibility, cell density, and electrical properties. Commonly, CTCs are larger than background blood cells, have greater density and weaker deformability and depict stronger charge than the remaining blood cells [83,84]. Hence, surface marker-independent separation techniques can be grouped into different categories, such as size-based, hydrodynamic, acoustophoretic, or electrokinetic methods [42,85,86,87]. Within hydrodynamic strategies, inertial microfluidics systems such as spiral microfluidics have emerged as efficient technologies for sized-based CTCs extraction, relying on hydrodynamic forces and channels geometry [88].

The High Throughput Vortex Chip (Vortex HT) has been proposed for size-based enrichment of CTCs. The device performs under high processing speeds with whole or diluted blood, presenting high purity in CTCS enrichment, with an average of 28.8 ± 23.6 background white blood cells per mL of whole blood. The chip was tested with stage IV lung and breast cancer patients demonstrating a capture efficiency up to 83%. Isolated CTCs were stained for CK and CK^-^ samples (40.8%) were additionally stained for VIM, N-Cadherin (NCAD), and EpCAM. Of those, 12.5% stained positive for VIM/NCAD, with 32.7% of CK+ cells staining positive for both mesenchymal and epithelial markers, whereas 42% were negative for all EMT markers, highlighting the importance of specific surface markers discovery and establishment [85].

Other methods based on acoustic, dielectrophoretic or magnetophoretic separation have also been reported [89,90]. Dielectrophoretic-based systems (DEP) can be an advantageous technique to gently isolate viable and intact CTCs and reduce the risk of clogging. However, considering that DEP separation systems rely on cells polarization differences, if any cell exhibits damaged membrane, dielectric differences may influence isolation, and damaged CTCs fail to be detected whereas a portion of WBC can be retained and not depleted, leading to low purity. Also, WBCs and CTCs may present similar dielectric properties, influencing purity isolation as well [91,92].

Although significant improvements in physical separation techniques have been achieved in the last years, there are still considerable challenges to overcome, as cell contamination, tumor cells heterogeneity, and lack of specificity. As each type of cells present in peripheral blood display cellular sizes within a considerable value range, overlapping sizes from different types of cells impact the performance of the devices, as well as in its results. Moreover, in size-based platforms, clogging is still a concern when it comes to membrane-based microfluidic systems using peripheral blood for CTCs extraction. Cells can accumulate and obstruct membrane’s pores/gaps, affecting the recovery efficiency of the platform. Additionally, due to high flow rates or because of cell extrusion, cells may not maintain their viability and integrity and commonly exhibit mechanical damage after isolation [69,93,94].

The combination of different label-free principles in a single microfluidic chip have shown to improve separation performance, enabling the detection of a wider range of tumor cells exhibiting different properties among them. For example, Wang et al. reported an inertial-based microfluidic device for the separation of CTCs from LCa patients’ samples combined with an integrated membrane filter. After pre-separation based on hydrodynamic forces in the doble spiral microchannel, CTCs are filtrated by a membrane filter with pore sizes of 8 μm. The inertial and sized-based separation system recovered 74.4% of spiked LCa CTCs [42].

Other researchers have also focused in developing microfluidic technologies based on multiple complementary isolation principles [95]. Most of these novel isolation methods employ pre-enrichment and isolation steps. Commonly, label-independent strategies are used as pre-enrichment step for continuous tumor cell enrichment. For subsequent isolation step, both label-dependent and label-free enrichment principles can be applied, continuously or discontinuously. Immunomagnetophoresis and immunocapture have been used for non-continuously recovering the tumor cells, while DEP, crossflow and immunomagnetophoresis have been applied for continuous CTC isolation. Immunomagnetophoresis, due to diverse options of usage for magnetic beads, might be employed for both retrieval manners [96,97,98].

CTC-iChip technology is a microfluidic system that uses three different antigen-independent principles for CTC isolation: deterministic lateral displacement, inertial focusing, and magnetophoresis. The system was tested with blood samples with tumor cells from diverse origins, achieving impressive isolation efficacies of about 97% [99].

### 4.2. Microfluidic-Assisted Strategies in ctDNA Research

Over the past years, several targeted approaches such as next generation sequencing (NGS), beads, emulsion, amplification, and magnetics (BEAMing) and digital PCR (dPCR) have allowed for the quantification of low frequency alterations in ctDNA with increased sensitivity [100]. However, the major obstacle for implementation in clinical routine is the lack of standardization for optimal DNA extraction. Miniaturization of these processes into a single chip may allow for simple and faster methods for extraction of nucleic acids. Thus, several lab-on-a-chip platforms designed to standardize ctDNA detection and characterization have been reported [101,102,103]. An overview on current microfluidic technologies for cfDNA isolation and analysis has been reported by Xu et al. [104].

Various solid phase extraction microdevices have been developed based on functionalized surfaces, silica membranes, or beads to bind and capture nucleic acids (Figure 2). Wu et al. reported on a solid phase extraction system comprising a monolithic tetramethyl orthosilicate-based sol-gel porous matrix that provided large surface area for DNA extraction. The system disclosed reproducible DNA EXTRACTION efficiency of about 70% for human blood [105].

A lab-on-a-disc with amine groups bond was developed by Jin et al. This system, composed by a dimethyl dithiobispropionimidate (DTBP)-based microchannel platform, enabled cfDNA capture in 15 min and with residual cellular background. The platform was compared to the spin-column method and results showed to be highly concordant. Additionally, KRAS and BRAF hot-spot mutations were identified using the DTBP platform, with >71% correlation between tissue and frozen plasma samples of 30 colorectal cancer patients. In some cases, KRAS mutations were detected in plasma of negative tissue samples [106].

Campos et al. developed a solid-phase extraction microfluidic device (µSPE) of plastic chips activated through UV/O3 via surface-confined carboxylic acid functionalities. An immobilization buffer (IB) composed of polyethylene glycol (PEG) and salts induce cfDNA condensation onto the activated surface of the microfluidic chip. The amount of cfDNA isolated was dependent on the density of carboxylate groups on the surface, and the cfDNA size on PEG and ethanol concentration and salt composition. The device showed promise not only in the recovered amount of cfDNA fragments (100–700 bp, >90%), even short ones (50 bp, >70%), but also in detecting KRAS mutation from plasma sample of NSCLC patients [107].

Magnetic beads are also used in DNA isolation. These particles are commonly exploited for their large surface area to volume ratio, easy manipulation under a magnetic field either in a stationary or laminar liquid flow, and adjusted surface modifications. Silica or cationic polymers are common coatings applied to functionalize the surface of these particles, since they are fitted to interact with negatively charged ctDNA. If the target DNA sequence is known, beads can also be functionalized with an oligonucleotide sequence like an aptamer [108,109,110,111].

Liquid phase extraction chips can rely on the mobility of nucleic acids via electrophoresis principles (EP) or chemical solutions (Figure 2). In EP microdevices, charged molecules move toward the electrically opposed particles according to direct current electric field [112,113]. In dielectrophoresis (DEP) microchips, charged particles are dielectric or uncharged and move when non-uniform electric field is implicated [114].

Manouchehri et al. first described a novel dielectrophoresis microarray chip to extract circulating cfDNA from the plasma of 12 chronic lymphocytic leukemia (CLL) patients with a processing time of 20 min. The system proved effective in detecting specific cancer mutations in SF3B1, NOTCH1, and TP53 genes in five samples, with 25 µL of plasma [115].

A pressure immiscibility-based extraction (PIBEX) novel microfluidic for centrifugation-free cfDNA extraction from blood plasma has been reported. The system contains a silica membrane and operates under a low vacuum pressure. The recovery rate of cfDNA and mutant fractions detected by this system was compared with the conventional gold standard (QIAamp circulating nucleic acid kit, QIAGEN) and depicted similar performance to the standard, while shortening the processing time without the repetitive centrifugation steps. When applied to the continuous monitoring of HER-2 type breast cancer, a point mutation in phosphatidylinositol-4,5-bisphosphate 3-kinase (PIK3CA) was detected, in a liver metastasis [116].

Sefrioui et al. presented a chip-based digital-PCR (dPCR) to detect and quantify cfDNA and ctDNA based on KRAS mutation of plasma from metastatic colorectal cancer patients. The detection rate of this mutated gene was evaluated by their platform and compared with TaqMan PCR (CAST-PCR). Results demonstrated a higher detection rate, of 69%, for the dPCR chip. Median OS was significantly decreased in patients with detectable ctDNA [117].

O’Keefe et al. reported on the development of the HYPER-melt (high-density profiling and enumeration by melt), a digital microfluidic approach for high-throughput molecular profiling. When applied for detection and assessment of intermolecular heterogeneity of DNA methylation, the device showed a sensitivity of detection as low as 1 methylated variant in 2 million unmethylated templates, of a tumor suppressor gene, CDKN2A (p14ARF). The microfluidic approach also showed a 20 to 300 times or more analytical sensitivity than the comparative quantitative methylation specific PCR (qMSP) assay in all methylation-positive samples of colorectal patients, when assessing NDRG4 methylation and detected it in two patients which had negative/nearly negative results by qMSP [118]. In their following work, the system was upgraded to a multilayer microfluidic device for efficient trapping and parallelized DNA methylation analysis of single molecules in picoliter-sized chambers. The platform was once again tested with methylated p14ARF by discriminating partially and fully methylated epialleles among a high background of unmethylated DNA and displayed an estimated loading efficiency of 80%, up to 7× more than the previous device, developing a simple and low-cost system for rare DNA methylation quantification [119].

## 5. Clinical Validation and Trials in Lung Cancer

Microfluidic technologies applied to liquid biopsies have achieved remarkable progresses in the past years, considering their relatively recent application to this field. Lab-on-a-chip platforms have become low-cost alternatives to commercially available kits, displaying faster processing times with miniaturization and automation, lower reagents consumption and waste production, offering highly sensitive and efficient recovery of biomarkers from several biofluids (blood, urine, saliva, and cerebrospinal fluid) [13,14,35].

Although these microchips may present several advantages compared to conventional laboratory methods for biomarkers enrichment and characterization, some challenges may cause setbacks in the technology implementation in clinical routine. An industrial scaling up of platforms developed for biomedical research may require higher expertise and training than the application of protocols or kits that use common laboratory and clinical equipment [14,120].

Problems that may be easily solved in bench-top research, by tuning certain processing parameters, may cause constraints in clinical practice, where standardized processes are used for patient’s evaluation. Nonetheless, the field has evolved rapidly with state-of-the-art technologies being introduced regularly. Problems such as air bubbles formation, leaking or precise flow controls that caused constraints in first generation microfluidics devices are not relevant at present, with cutting edge strategies of intelligent lab-on-a-chips incorporating automation and digitalization to on-chip single-cell analysis, miRNA detection and nucleic acids quantification and analysis [111,121,122,123,124]. However, the relatively new application of microfluidics technology for liquid biopsy translates into few clinical validation studies and trials involving microfluidic chips, which are especially scarce in the context of LCa.

Regarding CTCs, the only method approved by the US Food and Drug Administration (FDA) for clinical use is CellSearch^®^ system (Veridex), based on the enrichment of EpCAM-positive cells via immunomagnetic beads and applied for prognostication of breast, colon, and prostate cancer [125]. Remaining the only approved method for CTCs enrichment, CellSearch is commonly tested in other cancers of epithelial origin and used as a comparison method for CTCs isolation strategies.

In lung cancer, Krebs et al. compared the efficiency of CellSearch^®^ system with a label-free isolation by size of epithelial tumor cells technology (ISET, RareDiagnostics) for the assessment of 40 patients with stages III-IV NSCLC. CTCs were found in 80% of the patient samples using ISET versus 23% using CellSearch, with a subpopulation of cells isolated by ISET that did not express epithelial markers. In addition, clusters of more than 3 CTCs were detected in 43% of patients using ISET and were undetected by CellSearch [126].

Parsortix™ Cell Separation System (ANGLE), a semi-automated microfluidic based technology for CTCs isolation through size and deformability might be the first chip technology for CTCs enrichment approved by the FDA. The system is currently awaiting FDA clearance for its use in metastatic breast cancer patients. Parsortix has been tested also for label-free enrichment of CTCs in SCLC patients. CK positive CTCs were detected in all 12 patients enrolled in the study. Additionally, its efficacy was compared to CellSearch, in which CTCs were detected in 10 of the 12 patients, highlighting limitations of EpCAM-based capture in SCLC. The average number of recovered CTCs was also higher using the Parsortix system, for all 12 patient samples (average: >20 CTCs for Parsortix, >5 CTCs for Cellsearch) [127]. In NSCLC, the system was tested by Janning et al. to evaluate PD-L1 expression in CTCs of 127 patients. Results showed that an increase in PD-L1 positive CTCs might indicate resistance to immunotherapy [128].

Other lab-on-a-chip approaches for CTCs enrichment have been reported for clinical validation in lung cancer. A multi-flow microfluidics platform (MFM) for CTCs isolation in NSCLC was described by Zhou et al. The device displayed a recovery rate efficiency ranging from 87% to more than 93% in assays performed with cancer spiked cells. When applied to clinical samples, the device assisted in the detection of CTCs in 6 of 8 stage IV NSCLC 2 mL blood samples, with a median of 12 CTCs per mL of whole blood [129].

Xu et al. reported on an integrated microfluidic chip for clinical application in CTCs isolation and single cell analysis. Nineteen LCa patients were enrolled in this study, disclosing a CTC detection rate of 75%. Six somatic gene mutations were found by single cell analysis on a CTC from a patient with lung cancer. The results were confirmed by tissue biopsy analysis, showing the potential of singe-cell analysis application in CTCs research [130].

The graphene oxide (GO) chip has been employed for assessment of PD-L1 expression changes in CTCs after radiation therapy. The system consists of a microfluidic chamber and a substrate coated with GO nanosheets in which a cocktail of biotinylated antibodies was bound (anti-EpCAM, anti-CD133 and anti-EGFR). Changes in PD-L1 expression were monitored in 13 non-metastatic NSCLC patients. Results showed that PD-L1 is upregulated in CTCs during radiation therapy [23].

A collection of clinical trials on the use of CTCs for LCa are listed in Table 1. Different strategies have been employed in CTCs enrichment, with most of the selected studies resorting to microfluidic-based technologies.

By applying the ClearCell^®^ FX EP+ microfluidics chip, NCT02370303 trial (A Pilot Study to Isolate and Test Circulating Tumor Cells Using the ClearCell^®^ FX EP+ System) aimed at the isolation and quantification of circulating tumor cells for detection and treatment monitoring of LCa patients. The system consists in a spiral microfluidics that applies Dean Flow Fractionation principle to isolate CTCs from blood cells. Although 23 patients have been enrolled in this study, last updated in 2018, no results are available, yet.

Clinical trial NCT01193829 (Development of Circulating Tumor Cell Molecular Diagnostics Using a Novel Microfluidic Device) aimed at comparing EGFR mutations in primary NSCLC tumors of respective CTCs isolated via a label-free microfluidic device. Additionally, the clinical response of patients treated with gefitinib and its effect on CTCs EGFR mutations detected by the platform was also assessed.

Similarly, clinical trial NCT01734915 (Detecting EGFR T790M Mutations from Circulating Tumor Cells) was initiated in 2012 and intended to assess EGFR mutations in CTCs isolated using the microfluidic CTC-chip. The study aimed at comparing the EGFR mutation found in tissue biopsy and CTCs and matching genotyping, with the long-term goal of developing a less invasive liquid biopsy method to detect the EGFR mutation. In a resulting publication, the analysis of ctDNA was also described and compared to CTCs and tissue biopsies. CTCs were isolated with the CTC-chip device and lysed in situ for RNA and DNA extraction. T790M mutation was observed in 30 (75%) tumor biopsies, 28 (70%) isolated CTCs samples, and 32 (80%) ctDNA samples, with the authors reporting on mostly comparable results between CTCs/ctDNA and tissue-based genotyping.

Interestingly, while CTCs and ctDNA genotyping showed to be unsuccessful in 20 to 30% of the cases, the combination of both assays enabled genotyping of all patients, even identifying the T90M mutation in 35% of patients in whom tissue biopsy was negative or indeterminate, and further demonstrating the usefulness of combining circulating biomarkers characterization [131]. A few clinical trials combining the assessment of both biomarkers in LCa are also currently being performed (NCT04315753; NCT03771404).

Although liquid biopsies research initially focused on CTCs as the primary source for tumor-derived material, the discovery and growing interest in other biomarkers has led to increased research in point-of-care strategies for isolation and characterization of other circulating tumor material, such as cell free DNA. Whereas clinical trials on cell-free DNA in LCa are more recent (Table 2), strategies for cfDNA characterization in NSCLC have already been implemented in the clinical setting, contrary to CTCs. The Cobas^®^ EGFR Mutation Test v2 was the first method approved by the FDA, a PCR-based liquid biopsy test to detect EGFR mutations in NSCLC. In vitro diagnostics (IVD) tests to detect activating and resistance EGFR mutations in plasma, using allele-specific real-time PCR assays are currently employed and recommended for cfDNA EGFR testing to complement tissue biopsy or as an alternative when tumor tissue is limited or non-accessible [129]. While these methods disclose high specificity, sensitivity remains relatively low with false negatives being reported, as circulating tumor DNA may not express EGFR mutations in all patients whose tumors harbor mutations [132,133].

The ASSESS clinical trial (NCT01785888) performed an international, multicenter, and non-comparative study to evaluate the utility of plasma ctDNA in EGFR mutation testing in NSCLC patients with locally advanced or metastatic disease, across Europe and Japan (Table 2). Total of 1288 patients were enrolled in the trial. Different commercially available kits were used for cytology and plasma mutation analysis. Overall sensitivity of plasma mutation tests was 46%. EGFR mutation was detected in 16% of tissue and 9% of total plasma samples. False negative results were detected in 102 patients and 25 patients disclosed EGFR mutation positive result only in plasma. Mutation concordance between plasma and tissue samples was 89%, in 1162 matched pairs, with 97% specificity, 46% sensitivity, 78% positive predictive value, and 90% negative predictive value [133].

Probably the most widely applied microfluidics method in ctDNA analysis is droplet digital PCR (ddPCR, BIORAD). A microfluidic chip assists in sample preparation by water-oil emulsion and partition of the PCR reaction into thousands of individual nanoliter-sized droplets so that amplification occurs in each individual droplet. This technology disclosed a lower limit of detection (LOD: 0.001%) than any other PCR-based methods and more precise and reproducible results [104,134].

Taking advantage of the ddPCR technology, Zheng et al. assessed the EGFR T790M mutation in circulating tumor DNA in the plasma of NSCLC patients receiving TKI treatment. Overall survival correlated with the initial TKI treatment and the T790M mutation status. Moreover, the mutation was found in 47% of patients with acquired TKI resistance, suggesting that this approach might be useful to monitor the evolution of the EGFR mutation on patients receiving treatment [135].

ddPCR ctDNA analysis has been employed by Zhang et al. in clinical trial NCT02418234, along with ARMS analysis, for detection of T90M mutation. The study focused on 307 patients with advanced or recurrent NSCLC that had progressed during EGFR-TKIs treatment. Both methods showed an overall concordance of 78.3%, with the authors reporting a higher sensitivity with ddPCR. The detection of T90M mutation in ctDNA had an influence on clinical prognosis and subsequent treatment with AZD9291 related to the longest patient survival [136].

To date, ddPCR appears to be the only chip-based technology applied in clinical trials regarding LCa ctDNA assessment. Likewise, clinical validation studies with other microfluidic-based methods applied for ctDNA assessment in LCa are still rare.

The μSPE microchip developed by Campos et al. composed by arrays of micropillars to detect ctDNA showed efficacy for clinical disease detection of KRAS mutation gene in cfDNA extracted from plasma samples of 3 NSCLC and 5 colorectal cancer patients with the device, with less processing steps than the commercial kits [107].

A lab-on-a-disc system for fully automated and fast (<30 min) isolation of cfDNA from whole blood has been recently reported. The system, composed by newly developed electromagnetically actuated and reversible diaphragm valves integrated on a disc, allows for cfDNA purification, with plasma separation from whole blood, DNA binding and elution through a custom-designed centrifuge system. The lab-on-a-disc ability to purify cfDNA from peripheral blood of NSCLC patients with EGFR L858R mutations was compared with a commercial kit (Qiagen QIAamp Circulating Nucleic Acid Kit), demonstrating the same performance. Additionally, a patient follow-up was performed were the occurrence of resistance to TKI treatment for EGFR mediated by T790M was detected earlier than with the second tissue biopsy, improving patient personalized treatment selection [137].

A table summarizing the microfluidic devices applied for CTCs and ctDNA extraction in lung cancer clinical validation, presented in this section, is available as supplementary material (Table S1).

## 6. Concluding Remarks and Future Perspectives

Advances in precision oncology can guarantee a more thorough and complete characterization of the tumor, providing patients improved diagnostics, therapeutic strategy selection, and personalized follow-up. Especially for LCa, in which late diagnosis and limited-efficacy treatments continue to negatively influence patient survival, liquid biopsies can complement tissue biopsies and significantly improve personalized care. However, most of currently available isolation methods require complex protocols, with time-consuming and manual procedures that can result in less standardized results.

In general, many aspects may dictate the efficacy assessment of different liquid biopsy methods. Aside from the challenges of comparing different methods and kits based on the use of different laboratory equipment’s and practices, other variable conditions such as the sample amount, collection tubes, temperature, and time of sample storage can all influence the outcomes in biomarkers isolation.

Microfluidic based technologies have improved considerably since their first application to liquid biopsies. New integrated chips have shown striking results in biomarkers isolation, with rapid and more automated low-cost alternatives while presenting high sensitivity and specificity. Lab-on-a-chip systems can reduce potential errors in sample handling with fewer processing steps, closed architecture to minimize sample contamination or loss, and improve reproducibility, with easy high-scale production.

Although the field has been rapidly evolving, there are still aspects to improve before wide clinical routine implementation can be considered. Systems should present highly standardized and automatized results to appeal to the change from traditional methods. Additionally, most of current strategies studied for LCa are still under development with clinical validation phases comprising small sample size, whereas large patient cohorts are essential to establish device efficacy, sensitivity, and specificity. Specifically, for lung cancer, point-of-care strategies should consider the availability of the different biomarkers according to subtype and stage, as well as the mutational profile.

An optimal approach in which microfluidics can play a major role is combined assessment of biomarkers. Whereas current protocols and commercially available kits are designed specifically for the evaluation of one biomarker, microfluidics operation modes can easily allow for sequential retrieval of more than one biomarker from the same sample. CTCs and ctDNA may have complementary roles, considering their advantages and limitations. Whereas CTCs allow for the study of whole cells, DNA and RNA molecular profiling as well as protein expression, ctDNA can assess mutations, DNA methylation and copy number aberrations, with both supporting patient stratification, understanding and estimation of metastasis, estimate OS, characterize tumor’s mutational profile with real-time monitoring of disease progression and response to therapies, as well as detection of MRD.

Undoubtedly, lab-on-a-chip approaches are expected to play a pivotal role in liquid biopsies in the coming years. Microfluidics could assist in standardizing methods for cost-effective and fast tumor detection in at-risk patients, analysis of intratumoral and intertumoral heterogeneity and significantly improve LCa research, patient diagnosis, and clinical management of the disease.

## Figures and Tables

**Table 1:** Clinical trials regarding circulating tumor cells in lung cancer.

Name/NTC	Status	Condition/Patients (*n*)	Brief Summary
Development of Circulating Tumor Cell Molecular Diagnostics Using a Novel Microfluidic Device NCT01193829	Completed (2014)	NSCLC *n* = 30	To compare EGFR mutations between NSCLC and corresponding CTCs isolated by a label-free microfluidic device-based system. The device was tested for the feasibility to detect clinically relevant EGFR mutations in CTCs.
Detecting EGFR T790M Mutations from Circulating Tumor Cells NCT01734915	Completed (2016)	NSCLC with EGFR mutation *n* = 40	To determine whether the EGFR mutation can be detected in CTCs and if it reliably compares to tissue biopsy results. The CTC-chip was applied to isolate CTCs and identify the EGFR mutation in a less invasive way, aiming at facilitating the diagnosis of lung cancer.
A Study to Isolate and Test Circulating Tumor Cells Using the ClearCell^®^ FX EP+ System NCT02370303	Completed (2018)	Stage IV LCa Stage IB or higher NSCLC *n* = 23	To isolate and quantify CTCs of lung cancer patients using the ClearCell^®^ FX EP+ System, to advance cancer detection and treatment monitoring. For this, both blood and tumor samples were evaluated. Additionally, postoperative CTCs levels were assessed.
PDL-1 Expression on Circulating Tumor Cells in Non-small Cell Lung Cancer (IMMUNO-PREDICT) NCT02827344	Recruiting	Stage IV NSCLC *n*= 200	To demonstrate the feasibility of the analysis of PD-L1 expression on CTCs isolated by ISET filtration module.
Circulating Tumor Cells in Lung Cancer Screening (AIR) NCT02500693	Unknown	High-risk patients *n* = 600	To evaluate the predictive value of CTC detection for the diagnosis of lung cancer with the ISET technology.
Circulating Tumor Cells Spillage After Pulmonary Biopsy NCT02507778	Unknown	LCa *n* = 40	To quantify the number of CTCs and correlate with the tumor response to chemotherapy. Here, the CellCollectorTM, which detects and isolates EpCAM+ CTCs is applied.
Application of Detecting Circulating Tumor Cells in the Accurate Treatment of Early Stage Lung Adenocarcinoma (CTCs detection) NCT02951897	Unknown	Stage I Adenocarcinoma *n* = 120	To explore whether CTCs detection in patients diagnosed with early-stage lung cancer, and prior to surgery, can aid with early diagnosis or contribute to predict the prognosis and treatment strategies. The CanPatrolTM technology is applied for CTCs enrichment.
The Method ISET (Insulation by Size of Epithelial Tumor Cells) NCT00818558	Unknown	NSCLC *n* = 520	To evaluate the potential of ISET method to preoperative detection of CTC in patients with NSCLC. Furthermore, to correlate the presence of CTCs with pTNM stage, histology, and primary tumor cellularity.

Information collected from www.clinicatrials.gov, accessed on 5 January 2021. Abbreviations: LCa—lung cancer; NSCLC—non-small cell lung cancer; EGFR—epidermal growth factor receptor; CTCs—circulating tumor cells; ISET—isolation by size of epithelial tumor cells; pTNM—pathological tumor-node-metastasis; cfDNA—circulating cell-free DNA.

**Table 2:** Clinical trials regarding circulating cell-free DNA/circulating tumor DNA in lung cancer.

Name/NTC	Status	Condition/Patients (*n*)	Brief Summary
Europe-Japan Diagnostic Study for EGFR Testing (ASSESS) NCT01785888	Completed (2016)	Stage IIIA/B or metastatic NSCLC *n* = 1311	To assess the concordance of EGFR mutation status derived from tumor samples and blood-based cfDNA.
T790M Mutation on ctDNA in Patients with NSCLC After EGFR-TKI Failure NCT02418234	Completed (2018)	Metastatic/stage III NSCLC *n* = 314	To compare the frequency and abundance of T90M mutation using ARMS and ddPCR among different clinical modes of NSCLC patients with EGFR-TKI failure.
Study to Evaluate Concordance of Detecting EGFR Mutation by Circulating Tumor Free DNA Versus Tissues Biopsy in NSCLC. NCT03562819	Completed (2019)	Adenocarcinoma *n* = 269	To assess EGFR mutation status by circulation tumor free DNA in advanced NSCLC patients comparing to adenocarcinoma histology.
LIquid BIopsies in Patients Presenting Non-small Cell Lung Cancer (LIBIL) NCT02511288	Recruiting	Stage IIIB/IV NSCLC *n* = 900	To characterize the genetic profile of patients with advanced stage NSCLC through ctDNA using ddPCR and targeted NGF, whole genome sequencing.
Evaluation of the Feasibility and Clinical Relevance of Liquid Biopsy in Patients with Suspicious Metastatic Lung Cancer (LIBELULE) NCT03721120	Recruiting	Metastatic LCa *n*= 286	To perform genomic analyses of cfDNA using the InVision^®^ technology, by profiling the presence of genomic aberrations in a panel of 35 genes, including all actionable alterations required to initiate the appropriate first-line therapy (EGFR, ALK, ROS1, and BRAF V600E).
TR(ACE) Assay Clinical Specimen Study NCT02934360	Unknown	NSCLC *n* = 450	To evaluate the TR(ACE) Assay as a method of diagnostic, monitoring disease progress and response to therapy. This assay is a quantitative in vitro diagnostic test run in the TR(ACE) instrument that uses an electrokinetic technique to selectively capture cfDNA and others cellular debris directly from a blood sample.

Information collected from www.clinicatrials.gov, accessed on 5 January 2021. Abbreviations: cfDNA—circulating cell-free DNA; ctDNA—circulating tumor DNA; ARMS—amplification refractory mutation system; ddPCR—digital droplet PCR; EGFR—epidermal growth factor receptor; LCa—lung cancer; NSCLC—non-small cell lung cancer; TKIs—tyrosine kinase inhibitors.

## Supplementary Materials

The following are available online at https://www.mdpi.com/article/10.3390/cancers13092101/s1, Table S1: Overview of microfluidics-based methods for CTCs and cfDNA/ctDNA isolation in LCa clinical samples presented in Section 5. Clinical validation and trials in lung cancer.
