# Supplementary material for: Emerging Lab-on-a-Chip Approaches for Liquid Biopsy in Lung Cancer: Status in CTCs and ctDNA Research and Clinical Validation
Source: Cancers (Basel). 2021 Apr 27;13(9):2101. doi: 10.3390/cancers13092101 (PMC8123575; doi:10.3390/cancers13092101)
Supplement: Supplementary file 1 [file cancers-13-02101-s001.zip › cancers-1159519-supplementary.pdf]

cancers

MDPI

Review

Emerging Lab-on-a-Chip Approaches for Liquid Biopsy in Lung Cancer: Status in CTCs and ctDNA Research and Clinical Validation

Ângela Carvalho [1,2,3,*], Gabriela Ferreira [1,2,3], Duarte Seixas [1,2,3,4], Catarina Guimarães-Teixeira [3,4], Rui Henrique [3,4,5,6] Fernando J. Monteiro [1,2,3,7] and Carmen Jerónimo [3,4,6]

1 i3S-Instituto de Investigação e Inovação em Saúde, Universidade do Porto, Rua Alfredo Allen 208, 4200-135 Porto, Portugal; angela.carvalho@ineb.up.pt (A.C.); up201505325@fe.up.pt (G.F.); up201406215@fe.up.pt (D.S.); fjmont@ineb.up.pt (F.J.M.)
2 INEB-Instituto de Engenharia Biomédica, Universidade do Porto, Rua Alfredo Allen 208, 4200-135 Porto, Portugal
3 Porto Comprehensive Cancer Center (P.CCC), R. Dr. António Bernardino de Almeida, 4200-072 Porto, Portugal
4 Cancer Biology and Epigenetics Group, IPO Porto Research Center (GEBC CI-IPOP), Portuguese Oncology Institute of Porto (IPO Porto), R. Dr. António Bernardino de Almeida, 4200-072 Porto, Portugal; catarina.guimaraes.teixeira@ipoporto.min-saude.pt (C.G.-T.); rmhenrique@icbas.up.pt (R.H.); carmenjeronimo@ipoporto.min-saude.pt (C.J.)
5 Department of Pathology, Portuguese Oncology Institute of Porto (IPO Porto), R. Dr. António Bernardino de Almeida, 4200-072 Porto, Portugal
6 Department of Pathology and Molecular Immunology, Institute of Biomedical Sciences Abel Salazar, University of Porto (ICBAS-UP), Rua Jorge Viterbo Ferreira 228, 4050-513 Porto, Portugal
7 Faculdade de Engenharia, Departamento de Engenharia Metalúrgica e Materiais, Universidade do Porto, Rua Dr Roberto Frias, s/n, 4200-465 Porto, Portugal
* Correspondence: angela.carvalho@ineb.up.pt; Tel.: +351-226-074-900

Citation: Carvalho, Â.; Ferreira, G.; Seixas, D.; Guimarães-Teixeira, C.; Henrique, R.; Monteiro, F.J.; Jerónimo, C. Emerging Lab-on-a-Chip Approaches for Liquid Biopsy in Lung Cancer: Status in CTCs and ctDNA Research and Clinical Validation. *Cancers* **2021**, *13*, 2101. https://doi.org/10.3390/cancers13092101

Academic Editors: Federico Cappuzzo, Eriseld Krasniqi and Daniele Marinelli

Received: 10 March 2021
Accepted: 25 April 2021
Published: 27 April 2021

Publisher's Note: MDPI stays neutral with regard to jurisdictional claims in published maps and institutional affiliations.

Copyright: © 2021 by the authors. Licensee MDPI, Basel, Switzerland. This article is an open access article distributed under the terms and conditions of the Creative Commons Attribution (CC BY) license (http://creativecommons.org/licenses/by/4.0/).

Table S1. Overview of microfluidics-based methods for CTCs and cfDNA/ctDNA isolation in LCa clinical samples presented in section *5*. Clinical validation and trials in lung cancer.

Circulating Tumor Cells (CTCs)

System or Method	Cancer Type	Biofluid	Volume	Processing Time	Patients (*n*)	Main Outcomes	Reference
Parsortix™ Cell Separation System	SCLC	Whole blood	10 mL	Approx. 2 mL/h	12	**Range:** 20—1474 CTCs/ 7.5 mL (*n* = 12)	[128]
Parsortix™ Cell Separation System	NSCLC	Whole blood	7.5 mL	N/A	97	**Median:** 2 CTCs **Range:** 1—54 CTCs (*n* = 59)	[129]
Multi-flow microfluidics platform (MFM)	NSCLC	Whole blood	2 mL	N/A	8	**Median:** 12 CTCs/mL **Maximum:** 78 CTCs/mL (*n*= 6)	[130]
Integrated microfluidic platform	NSCLC	Whole blood	4 mL	30 min	20*	**Detection rate:** 75% (*n* = 15)	[131]
The graphene oxide (GO) chip	NSCLC	Whole blood	1 mL	1 mL/h	13†	**Average:** 21.3 CTCs/ml **Range:** 4–72 CTCs/ml (*n* = 38)	[23]

* 19 NSCLC patients and 1 gastric cancer patient, † 3 independent collections from each patient—total of 38 samples.

Circulating cell-free DNA (cfDNA)/ Circulating tumor DNA (ctDNA)

System or Method	Cancer Type	Biofluid	Volume	Processing Time	Patients (*n*)	Main Outcomes	Reference
μSPE microchip	NSCLC	Plasma	up to 5 mL	2 μl/min or 0.8 μl/min	3	**Median:** 1.0 μg/mL **Range:** 0.222–9.3 μg/mL (*n* = 3) KRAS mutations (n=2)	[108]
Fully automated Lab-on-a-disc	NSCLC	Whole blood	>3 ml	<30 min	15	L858R mutation detection (*n* = 9)	[138]

Abbreviations: NSCLC- Non-small cell lung cancer; SCLC—Small cell lung cancer; CTCs—Circulating tumor cells.
